# Antioedematous and Analgesic Properties of Fertile Fronds of *Drynaria quercifolia*

**DOI:** 10.1155/2014/302089

**Published:** 2014-01-20

**Authors:** G. I. Anuja, P. G. Latha, V. J. Shine, S. R. Suja, P. Shikha, K. Satheesh Kumar, S. Rajasekharan

**Affiliations:** ^1^Ethnomedicine and Ethnopharmacology Division, Jawaharlal Nehru Tropical Botanic Garden and Research Institute, Palode, Thiruvananthapuram 695562, India; ^2^Biotechnology and Bioinformatics Division, Jawaharlal Nehru Tropical Botanic Garden and Research Institute, Palode, Thiruvananthapuram 695562, India

## Abstract

Inflammation is a complex biological response of tissue cells to harmful stimuli including trauma, tissue necrosis, and infections which plays a key role in the pathophysiology of many deadly diseases. In ethnomedicine *Drynaria quercifolia* fronds are used to treat inflammation as poultice on swellings and as antibacterial, hepatoprotective, and antipyretic agent. Herein, we have evaluated the antioedematous, antiproliferative, and analgesic properties of the ethanolic extract of fertile fronds of *D. quercifolia* (FF) by standard procedures. Oral administration of FF produced significant inhibition of carrageenan and histamine induced paw oedema in Wistar rats. FF significantly reduced both wet weight and dry weight of granuloma tissue which shows the inhibitory effect on exudative and proliferative phases of inflammation. FF significantly attenuated acute and delayed phases of formalin induced pain, acetic acid-induced writhing, capsaicin-induced nociception, and hot plate test in mice. Phytochemical analysis revealed the presence of coumarins, flavonoids, glycosides, phenolics, saponins, steroids, tannins, and terpenoids. Total phenolic content was 186 mg/g equivalent of gallic acid. The HPLC estimation showed flavanone glycoside naringin (1.2%) and its aglycone naringenin (0.02%). The presence of potent anti-inflammatory and analgesic principles in FF and their synergistic action may be the reason for the proposed therapeutic effects.

## 1. Introduction

Inflammation is a complex biological response of tissue cells to harmful stimuli including mechanical trauma, tissue necrosis, and infections which plays a key role in the pathophysiology of many deadly diseases including Alzheimer's disease, Type 2 diabetes, rheumatoid arthritis, liver cirrhosis, cancer, and neurological, pulmonary, and cardiovascular diseases. Although several modern drugs are used to treat these types of disorders, their prolonged use may cause severe adverse side effects [1]. Consequently there is a need to develop new anti-inflammatory agents with minimum side effects [2]. Most of the present day analgesic drugs also exert a wide range of side effects [3]; a study on plant species that are traditionally used as pain killers must be adopted as a research strategy in the development of analgesic drugs [4]. Herbal drugs play a major role in the treatment of human ailments due to their safety, efficacy, and cost effectiveness [5]. Dependence on plants as a source of medicine is prevalent in developing countries where traditional medicine plays a major role in health care [6]. Infectious and inflammatory diseases are among those treated using traditional remedies [7].

*Drynaria quercifolia* (L.) J. Smith (Polypodiaceae) is an epiphytic/epilithic medicinal pteridophyte, distributed widely in the Western Ghats, and locally called “Marappannakizhangu” or “Attukalkizhangu.” Fertile fronds are long stalked, large, 2–8 ft. long, pinnately lobed, and leathery or membranous in texture. The fertile fronds bear the sori, which are round and superficial. The fronds are emollient, pectoral, and expectorant, used as poultice on swelling, and possess antibacterial property [8]. In Malaysia fronds are used as poultice on swellings [9]. The whole plant of *D. quercifolia* is anthelmintic and tonic, used to treat chest, skin diseases, and loss of appetite [10]. It is also used to treat jaundice and is used as a poultice, antifertility agent [11] and antipyretic agent [12, 13]. In the present study we have aimed to evaluate the antioedematous and analgesic properties of fertile fronds of *D. quercifolia* using *in vivo* pharmacological methods.

## 2. Materials and Methods

### 2.1. Chemicals

Acetyl salicylic acid (aspirin), carrageenan, gallic acid, histamine, indomethacin, naringin, naringenin, and sodium carbonate were obtained from Sigma Chemicals Co., USA. Sodium salicylate was purchased from Hi-Media, India, and thiosol sodium from Neon Laboratory, India. All the other chemicals were of analytical and HPLC grade.

### 2.2. Plant Material

#### 2.2.1. Collection of Plant Material

The fertile fronds of *D. quercifolia* were collected from the Western Ghats and authenticated by plant taxonomist of the Institute. A voucher specimen had been deposited at the Herbarium of the Institute (TBGT 57025, dated August 1, 2012).

#### 2.2.2. Preparation of the Plant Extract

The fertile fronds were washed thoroughly with tap water, cut to 1 cm cubes, shade-dried, and powdered. The powder (100 g) was extracted with 95% ethanol (1000 mL) for 24 h, at room temperature with constant stirring. The extract was filtered and the filtrate was concentrated at 30°C under reduced pressure in a rotary evaporator. The yield (w/w) of the crude extract was found to be 6%. The crude extract was suspended in 1% Tween-80 to required concentrations and used for the experiments and referred to as FF.

### 2.3. Experimental Animals

Wistar rats (150–250 g) and Swiss albino mice (25–30 g) of either sex were selected for the study, housed in poly acrylic cages (two animals per cage), and maintained under standard laboratory conditions (temperature 24–28°C, relative humidity 60–70%, and 12 h light/dark cycles). They were fed commercial rat feed (Lipton India Ltd. Mumbai) and boiled water *ad libitum*. All experiments involving animals were done according to NIH guidelines, after getting the approval of the Institute's Animal Ethics Committee.

### 2.4. *In Vivo* Studies

#### 2.4.1. Carrageenan-Induced Paw Oedema in Rats

Rats were divided into 5 groups of 6 animals each. Group 1 (carrageenan control, CC) received p.o., 1% Tween-80, 30 min prior to carrageenan injection, groups 2, 3, and 4 were given 125, 250, and 500 mg/kg of FF p.o., and group 5, the standard group, was given p.o., as aqueous solution of indomethacin (10 mg/kg), respectively, 30 min prior to carrageenan injection. The paw volume was measured plethysmographically just before and 3 h after carrageenan injection. The percentage inhibition of oedema was calculated for each group with respect to the vehicle treated control group [14].

#### 2.4.2. Histamine Induced Paw Oedema in Rats

Anti-inflammatory activity of FF was assessed by the histamine-induced paw oedema method. Rats were divided into 5 groups of 6 animals each. Animals of all the groups were injected with 0.1 mL of 0.1% histamine in the right hind foot under the plantar aponeurosis. Group 1 animals (histamine control, HC) received p.o., 1% Tween-80, 30 min prior to histamine injection, groups 2, 3, and 4 were given 125, 250, and 500 mg/kg of FF p.o., respectively, 30 min prior to histamine injection, and group 5, the standard reference group, was given p.o., an aqueous solution of indomethacin (10 mg/kg), 30 min prior to histamine injection. The paw volume was measured using a plethysmometer just before and 1 h after histamine injection. The percentage inhibition of oedema was calculated for each group with respect to the vehicle treated control group [15].

#### 2.4.3. Cotton Pellet Granuloma Formation in Rats

The effect of FF on granuloma formation was studied in Wistar rats. The dorsum of the rats was shaved and wiped with alcohol. A 1 cm midline incision was made in the interscapular region. Sterile cotton pellets (100 ± 1 mg) were inserted on either side of a midline incision (1 cm) made on the dorsum of rats and the incision was closed by interrupted sutures. FF (125, 250, and 500 mg/kg) was administered p.o., daily for 7 consecutive days starting from the day of pellet implantation. Indomethacin (10 mg/kg, p.o.) was given to the standard group while the control group received p.o., an equal quantity of 1% Tween-80. The animals were sacrificed on the 8th day in which the cotton pellets were removed, cleaned of extraneous tissue, weighed and dried in hot air oven at 80°C for 24 h [16].

#### 2.4.4. Acetic Acid-Induced Writhing in Mice

Mice were divided into 5 groups (6 mice/each group). All the groups received i.p., 0.5% aqueous solution of acetic acid. Group1, the acetic acid control (AC) group, received a single dose of 1% Tween-80 (0.5 mL) p.o., 20 min prior to the administration of acetic acid. Groups 2, 3, and 4 received p.o., 100, 200, and 300 mg/kg of FF whereas group 5, the standard control group, received p.o., acetyl salicylic acid (aspirin) (100 mg/kg), respectively, 20 min prior to the administration of acetic acid. The number of writhes per animal was recorded during the 20 min period, beginning 5 min after the injection of acetic acid [17].

#### 2.4.5. Formalin Induced Paw Licking in Mice

Mice were divided into 5 groups of 6 animals each. Group 1, the control group, received 1% Tween-80, p.o., groups 2, 3, and 4 received 100, 200, and 300 mg/kg FF extract p.o. whereas group 5, the standard group received sodium salicylate (100 mg/kg), 30 min prior to formalin injection. 20 *μ*L of 1% formalin was injected into the dorsal surface of the left hind paw. The time spent for licking the injected paw was recorded. Animals were observed for the 5 min after formalin (acute phase) or for 10 min starting at 20 min after formalin (delayed phase) [18].

#### 2.4.6. Capsaicin-Induced Paw Licking in Mice

To provide direct evidence concerning the antinociceptive effect of FF on neurogenic nociception, FF (100, 200, and 300 mg/kg p.o.) was tested against capsaicin-induced paw licking in mice. Morphine 7.5 mg/kg (s.c.) was used as the standard. The animals were treated with 1% Tween-80 (vehicle), FF, and morphine and placed in a glass box. After 30 min (which served as an adaptation period) 20 *μ*L of capsaicin (1.6 *μ*g/paw) was injected into the right paw. Capsaicin was dissolved in 5% ethanol, 95% phosphate buffer saline, and pH 7.4. Animals were observed individually for 5 min following capsaicin injection. The amount of time spent licking the injected paw was recorded and considered indicative of nociception [19].

#### 2.4.7. Hot Plate Test in Mice

Analgesia of FF was assessed using Eddy's hot plate (thermal stimulus), temperature 55 ± 1°C. Mice were divided into 6 groups of 6 animals each. Group 1, the control group, received 1% Tween-80, p.o., groups 2, 3, and 4 received 100, 200, and 300 mg/kg FF extract, p.o., and groups 5 and 6 the standard groups received sodium salicylate (100 mg/kg) and morphine (7.5 mg/kg). Mice were placed on hot plate maintained at 55 ± 1°C at 0, 15, 30, 60, 90, and 120 min after drug administration. The time taken by animals to lick the fore or hind paw or jump off the plate was taken as the reaction time(s); 15 s cutoff was used to prevent tissue damage [20].

#### 2.4.8. Acute Toxicity Study

Six groups of 10 mice each were administered p.o., 500, 1000, 2000, 3000, 4000, and 5000 mg/kg, respectively, of FF, maintaining appropriate controls. All the animals were observed continuously for the first 3 h then 1 h intermittently up to 24 h for behavioral changes like convulsions, hyperactivity, sedation, grooming, loss of righting reflex, epilation, respiratory rate, food and water intake, state of fecal pellets, and mortality. The animals were observed for post treatment toxic symptoms daily for 7 days after treatment [21].

### 2.5. Phytochemical Analysis

FF was subjected to preliminary phytochemical analysis and TLC studies, as per the standard methods [22].

#### 2.5.1. Estimation of Total Phenolics in *D. quercifolia* Fertile Fronds Ethanolic Extract (FF)

The total phenolic content in FF was determined by Folin-Ciocalteu's method. A standard curve was prepared using different concentrations of gallic acid (10, 25, 50, 100, 110, 125, 150, and 200 *μ*g/mL) prepared in ethanol. To the above standard solutions of gallic acid (1 mL each), 5 mL of Folin-Ciocalteu's reagent (1 : 10 dilution in distilled water) was added and, after 8 min, 4 mL of 7.5% sodium carbonate was added. These solutions were incubated at room temperature for 2 h and their absorbances at 765 nm were measured on UV-visible spectrometer (UV-1650PC, Shimadzu, Japan). For the test solution, 1 mL of FF (10 mg in 10 mL EtOH) was mixed with 5 mL of Folin-Ciocalteu's reagent and 4 mL of 7.5% sodium carbonate was added after 8 min and incubated at room temperature for 2 h and its absorbance was also read at 765 nm. The gallic acid equivalent (GAE) was plotted and the total phenolic content of FF was calculated in terms of gallic acid equivalents by considering the dilution factor [23].

#### 2.5.2. High Performance Liquid Chromatography (HPLC) Analysis of *D. quercifolia* for the Detection of Naringin and Naringenin

The HPLC analysis of *D. quercifolia* was carried out on HPLC system (Gilson, France) driven by a UNIPOINT system software and the chromatographic separations were performed using a C18 Kromasil Column (250 × 4.6 mm), with a flow rate of 1 mL/m and a sample size of 15 *μ*L. The mobile phase used was methanol, acetonitrile, and ammonium acetate buffer (5 : 11 : 4) and isocratic elution was performed. The sample was monitored with UV detection at 260 nm at the flow rate of 1 mL min^−1^ at ambient temperature. Retention time for naringin was found at 2.3 min and that for naringenin was at 2.6 min [24].

### 2.6. Statistical Analysis

The results were expressed as mean ± standard deviation of mean (SD). Analysis of variance (ANOVA) was done to compare and analyse the data followed by Duncan's multiple range test. Effects were considered significant at *P* ≤ 0.01 [25].

## 3. Results

### 3.1. Effect of FF and Indomethacin on Carrageenan-Induced Paw Oedema in Rats

**Figure 1:**
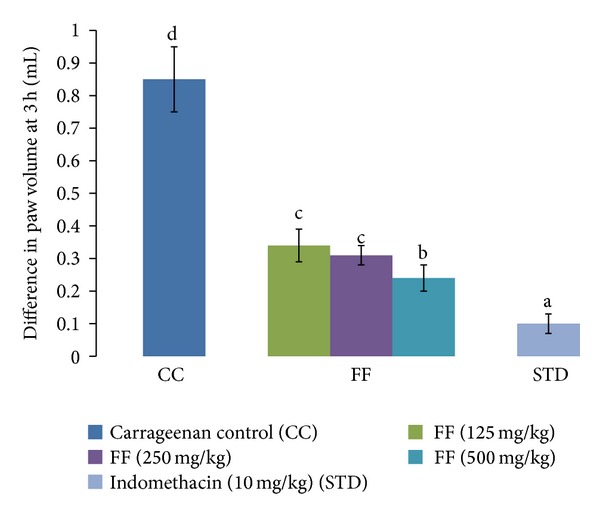
Effect of FF and indomethacin on carrageenan-induced paw oedema in rats. Results are expressed as mean ± SD. Values bearing different letters as superscripts showed significant differences (*P* < 0.01) using one-way ANOVA, Duncan's multiple range test.

FF at all the doses (125, 250, and 250 mg/kg) studied significantly inhibited the carrageenan-induced paw oedema in rats in a dose-dependent manner, 60.00%, 63.53%, and 71.76%, respectively. The standard group treated with indomethacin showed 88.24% inhibition of oedema formation (Figure 1).

### 3.2. Effect of FF and Indomethacin on Histamine Induced Paw Oedema in Rats

**Figure 2:**
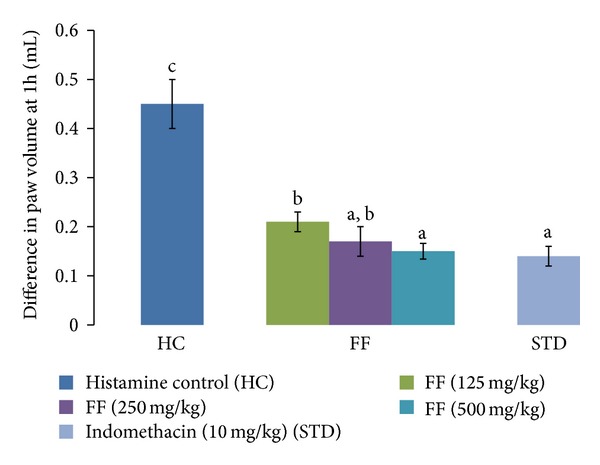
Effect of FF and indomethacin on histamine induced paw oedema in rats. Results are expressed as mean ± SD. Values bearing different letters as superscripts showed significant differences (*P* < 0.01) using one-way ANOVA, Duncan's multiple range test.

The amount of oedema produced was quantified 1 h after histamine injection. FF at the 3 doses (125, 250, and 500 mg/kg) studied significantly inhibited the histamine induced paw oedema in rats, 53.33%, 62.22%, and 66.67%, respectively. The group treated with indomethacin (10 mg/kg) showed 68.89% inhibition (Figure 2).

### 3.3. Effect of FF and Indomethacin on Cotton Pellet Granuloma Formation in Rats

**Figure 3:**
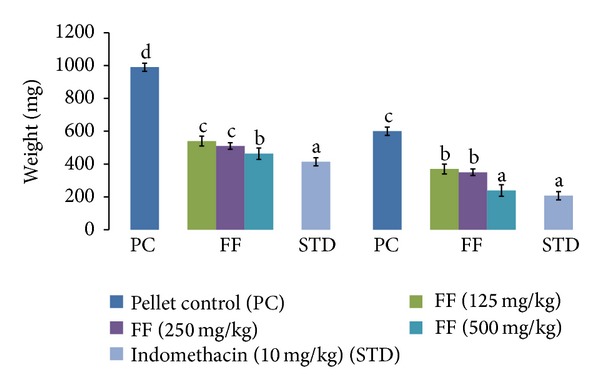
Effect of FF and indomethacin on the wet weight (exudative phase) and dry weight (proliferative phase) of cotton pellet induced granuloma in rats. Results are expressed as mean ± SD. Values bearing different letters as superscripts showed significant differences (*P* < 0.01) using one-way ANOVA, Duncan's multiple range test.

**Figure 4:**
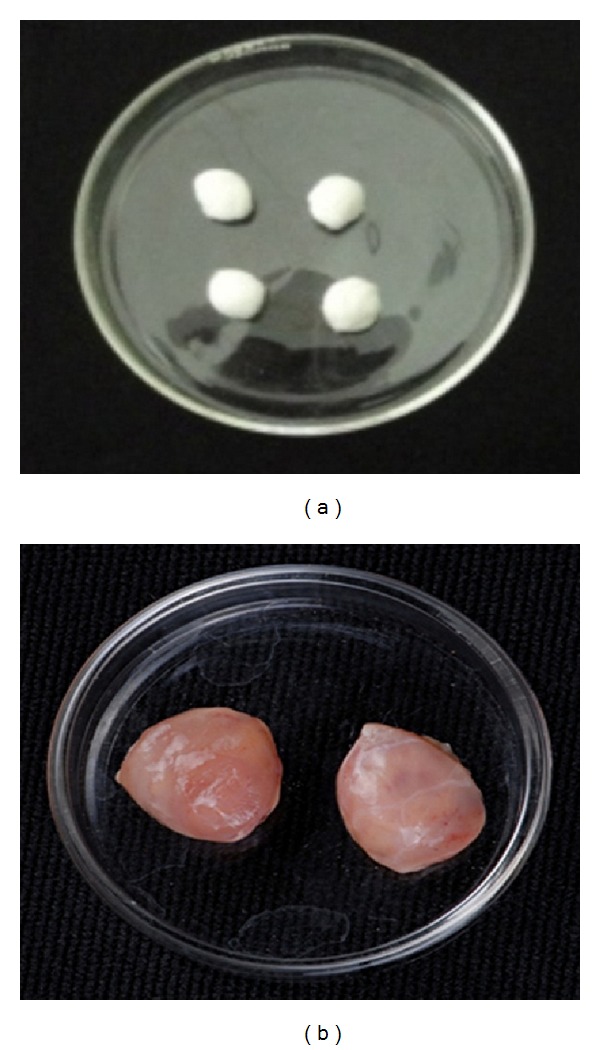
Cotton pellets and formation of granuloma tissue around cotton pellets implanted in rats after 7 days.

FF produced a significant dose-dependent inhibition of granuloma formation. It was almost equally potent as indomethacin in inhibiting both exudative and proliferative phases of granuloma formation. The formation of granuloma tissue around implanted cotton pellet is shown in Figure 3. FF at 500 mg/kg dose produced 53.21% inhibition in the exudative phase and 60.18% in the proliferative phase (Figure 3). The formation of granuloma tissue around implanted cotton pellet is shown in Figure 4.

### 3.4. Effect of FF and Aspirin on Acetic Acid-Induced Writhing

**Figure 5:**
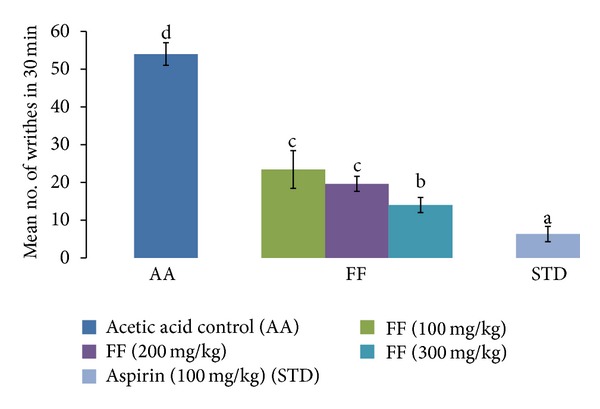
Effect of FF and aspirin on acetic acid-induced writhing in mice. Results are expressed as mean ± SD. Values bearing different letters as superscripts showed significant differences (*P* < 0.01) using one-way ANOVA, Duncan's multiple range test.

Intraperitoneal injection of acetic acid produced 54.0 ± 2.0 writhes in the control group, 20 min after injection. The groups pretreated with FF (100, 200 and 300 mg/kg) inhibited the number of writhes in a dose-dependent manner, 56.66%, 63.70%, 74.07%, respectively. Aspirin 100 mg/kg produced 88.33% inhibition of writhes (Figure 5).

### 3.5. Effect of FF and Sodium Salicylate on Formalin Test in Mice

**Figure 6:**
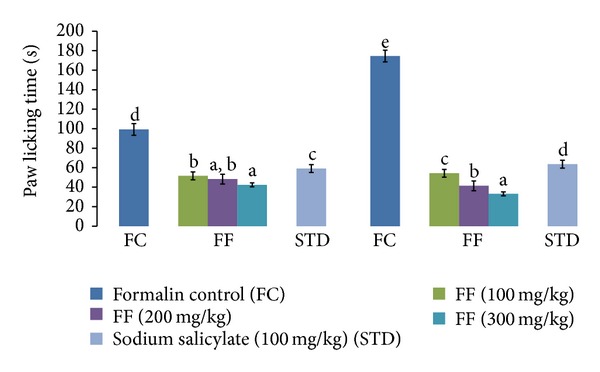
Effect of FF and sodium salicylate (SS) on formalin induced paw licking in mice. Results are expressed as mean ± SD. Values bearing different letters as superscripts showed significant differences (*P* < 0.01) using one-way ANOVA, Duncan's multiple range test.

SS did not show any significant effect on the acute phase of the formalin test (40.48%), but FF at 100, 200, and 300 mg/kg reduced the nociception in this phase in a dose-dependent manner (48.04%, 51.46%, and 57.30%). In the delayed phase of formalin test, SS produced only 63.55% inhibition while FF reduced the nociception significantly that is, 68.94%, 76.28%, and 81.03% dose dependently (Figure 6).

### 3.6. Effect of FF and Morphine on Capsaicin-Induced Paw Licking in Mice

**Figure 7:**
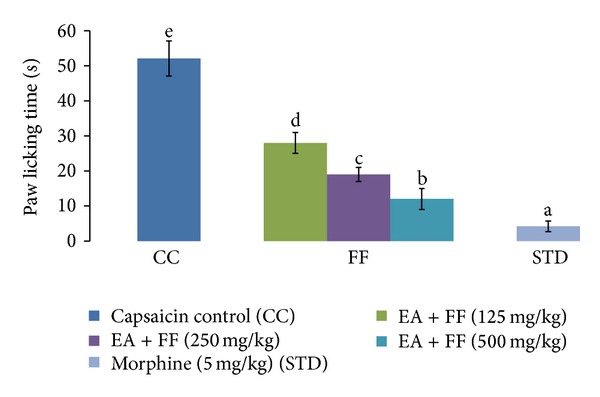
Effect of FF and Morphine on capsaicin-induced paw licking in mice. Results are expressed as mean ± SD. Values bearing different letters as superscripts showed significant differences (*P* < 0.01) using one-way ANOVA, Duncan's multiple range test.

Intraplantar injection of capsaicin (1.6 *μ*g) evoked nociceptive paw licking behavior in mice that appeared maximal up to 5 min. A significant decrease in the duration of paw licking was observed in mice pretreated with FF at doses of 100, 200, and 300 mg/kg (60.15%, 67.20%, and 71.33%). The results show that FF produced marked and dose-related inhibition of the capsaicin-induced neurogenic pain in mice. Morphine (7.5 mg/kg) was found to be very effective (91.94%) against capsaicin-induced pain (Figure 7).

### 3.7. Effect of FF on Hot Plate Test in Mice

In the hot plate study, FF significantly delayed the response of mice to hot plate thermal stimulation when compared to the vehicle treated control group. Maximum protection was produced at 60 min by FF at 300 mg/kg dose. Morphine was found to be highly effective in the hot plate test and offered better protection than sodium salicylate. The results suggest that FF may have central analgesic effect evidenced by the increase in the reaction time of drug treated mice in hot plate test as summarized in Table 1.

### 3.8. Effect of FF on Acute Toxicity Mice

In the present study, oral administration of FF up to 5 g/kg did not produce any behavioural changes. No lethality occurred up to the doses studied and the results of acute toxicity studies in mice indicated that the LD_50_ value is higher than 5 g/kg for FF.

### 3.9. Phytochemical Studies on FF

**Figure 8:**
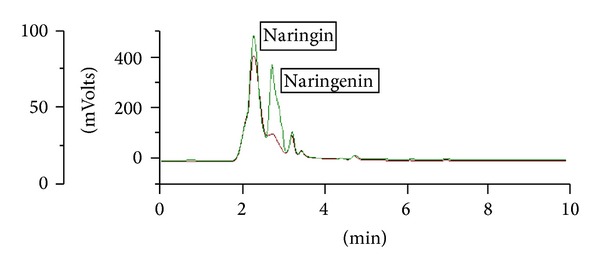
HPLC profile of FF showing the presence of naringin and naringenin with coinjection of both standards. The red line shows the extract and green line shows standard naringin with retention time 2.3 min and naringenin 2.6 min.

Preliminary phytochemical studies revealed the presence of phenolics, flavonoids, catechin, steroids, coumarins, saponins, tannins, and terpenoids in FF. The total phenolic content was found to be 186 mg/g equivalent gallic acid. HPLC studies revealed the presence of the flavanone glycoside naringin 1.2% and its aglycone naringenin 0.02% in fertile fronds (Figure 8).

## 4. Discussion

Oedema formation due to carrageenan in the rat paw is a biphasic event [26]. The first phase occurs within 1 h of injection and is partly due to the trauma of injection and also due to the release of histamine and serotonin. The second phase of oedema is due to the release of prostaglandins [27]. The carrageenan-induced paw oedema model in rats is known to be sensitive to cyclooxygenase inhibitors and has been used to evaluate the effect of nonsteroidal anti-inflammatory agents which primarily inhibit cyclooxygenase involved in prostaglandin synthesis [28]. Thus from the results of the present study, it may be inferred that the inhibitory effect of FF on carrageenan-induced inflammation in rats may be due to inhibition of cyclooxygenase leading to inhibition of prostaglandin synthesis.

Histamine is a mediator in the inflammatory pathway and FF significantly reduced the histamine induced paw oedema in rats in a dose-dependent manner. Earlier reports show that histamine increases vascular permeability during inflammation after injury [29] which leads to oedema formation. The reduction in the paw oedema after pretreatment with FF may be due to the suppression of increased vascular permeability caused by the extract.

The weight of the wet cotton pellets correlates with exudative material and the weight of dry pellets correlates with the amount of granulomatous tissue. The suppression of proliferative phase could result in decrease in the weight of granuloma formation [30] indicated by the decrease in the dry weight of cotton pellet granuloma which may be due to the suppression of the proliferative agents involved in formation of granuloma tissue [31, 32]. In the present study FF offered significant protection in both exudative and proliferative phases of cotton pellet induced granuloma.

The analgesic potential of FF has been evaluated using chemical as well as thermal stimuli induced pain models. Oral administration of FF was found to be effective on all the pain models studied. Acetic acid-induced writhing response in mice is a simple and reliable model to evaluate peripheral type of analgesic action of herbal drugs. The abdominal constrictions are related to the sensitization of nociceptive receptors to prostaglandins [33]. Acetic acid is reported to cause an increase in the peritoneal fluid level of prostaglandins and inflammatory pain by inducing capillary permeability. The results of the present study indicate that the analgesic effect of FF may possibly involve inhibition of prostaglandins.

The formalin test in mice is sensitive to nonsteroidal anti-inflammatory drugs and other mild analgesics. Formalin produces a distinctive biphasic response. The acute phase corresponds to acute neurogenic pain which reflects directly the effect of formalin on nociceptors and is sensitive to drugs that interact with the opioid system. Delayed phase corresponds to inflammatory pain responses and inhibited by analgesic and anti-inflammatory drugs [18]. Thus the nociceptive effect of FF on the first phase of formalin test proposes to its central action. The protective effect of FF on the second phase of formalin test is due to anti-inflammatory effect on peripheral tissue which explains the antinociceptive effect in the second phase. The results of the hot plate test also suggest that the central analgesic effect of FF and the analgesic action demonstrated by FF in hot plate test may involve supraspinal components.

Oral administration of FF elicited a dose-dependent antinociceptive effect on the capsaicin-induced neurogenic paw-licking response. Capsaicin (8-methyl-*N*-vanillyl-6-nonenamide), the pungent analgesic substance obtained from hot red chilli peppers, is regarded as a valuable pharmacological tool for studying a subset of mammalian primary sensory C-fibers and A*γ* afferent neurons including polymodal nociceptors and warm thermoreceptors [34]. It has been proposed that the capsaicin-induced nociception occurs as a result of the activation of the capsaicin (vanilloid) receptor, TRPV1, a ligand-gated nonselective cation channel present in primary sensory neurons [35, 36]. The reduction in this pain model suggests that FF is effective against neurogenic pain in mice and its action may be due to an interaction with the related receptor.

Moreover, we have already reported the anti-inflammatory and analgesic properties of *D. quercifolia* rhizome [37]. Our findings reveal that both the rhizomes and the fertile fronds possess significant anti-inflammatory and analgesic properties.

The efficacy of herbal remedies is attributed to various active constituents in combination. In the recent studies, with plant drugs, it is reported that polyphenols possess anti-inflammatory and analgesic effects [38]. The antinociceptive properties of many plants have been attributed to the flavonoids [39, 40], tannins [41], triterpenes [40, 42], and coumarins [43]. Terpenes and diterpenoids are intensively investigated because they counteract acute and chronic inflammation and pain [19, 44–48]. Herein, the presence of potent anti-inflammatory and analgesic principles in FF like coumarins, flavonoids, glycosides, phenolics, saponins, steroids, tannins, terpenoids, flavanone glycoside naringin, and its aglycone naringenin is reported. Kamboj [49] also reported the presence of 0.067% naringin in the methanol extract of fronds of *D. quercifolia*.

The anti-oedematous property of FF may be due to the inhibition of proinflammatory mediators, free radical scavenging, or membrane stabilizing effects. The proposed analgesic property of FF may be due to its effect on peripheral nociceptors, spinal mediated central action, or interaction with various receptors including capsaicin receptors. The synergistic action of the phytochemicals present in FF could be the reason for the proposed anti-inflammatory and analgesic effects. However, detailed studies are warranted to decipher the exact nature and mechanism of action of the phytochemicals responsible for the therapeutic effects of FF.

## 5. Conclusion

The results obtained from the present study reveal that *Drynaria quercifolia* fertile frond ethanolic extract (FF) has potent antioedematous and analgesic properties which substantiates its ethnomedicinal claims in the treatment of inflammation and pain.

## Figures and Tables

**Table 1:** Effect of ethanolic extract of fertile fronds (FF), sodium salicylate (SS), and morphine on hot plate test in mice.

Treatment groups	Time taken to respond (s)					
	0 min	15 min	30 min	60 min	90 min	120 min
Vehicle control	5.40 ± 0.6^a^	5.52 ± 1.3^a,b^	5.83 ± 1.3^a^	7.10 ± 1.5^a^	7.30 ± 1.1^a^	7.42 ± 1.2^a^
FF (100 mg/kg)	5.32 ± 1.2^a^	5.53 ± 1.1^a,b^	7.86 ± 0.6^b^	8.18 ± 1.2^a,b^	8.26 ± 1.2^b,c^	8.30 ± 1.0^b^
FF (200 mg/kg)	5.56 ± 1.2^b^	5.64 ± 1.3^b^	8.25 ± 1.2^b,c^	8.40 ± 1.1^b^	8.90 ± 0.6^c^	9.12 ± 1.1^c^
FF (300 mg/kg)	5.31 ± 0.7^a^	5.82 ± 1.5^b,c^	9.10 ± 1.1^c^	10.4 ± 1.2^c^	10.8 ± 1.5^d^	11.25 ± 1.1^d^
SS (100 mg/kg)	5.47 ± 1.6^b^	5.10 ± 1.0^a^	8.10 ± 1.5^b,c^	8.70 ± 0.8^b^	8.85 ± 1.1^c^	8.08 ± 1.4^b^
Morphine (7.5 mg/kg)	5.22 ± 1.1^a^	6.80 ± 1.2^c^	10.81 ± 1.1^d^	14.7 ± 1.6^d^	15.5 ± 1.1^e^	16.15 ± 1.1^e^

Results are expressed as mean ± SD. Values bearing different letters as superscripts showed significant differences (*P* < 0.01) using one-way ANOVA, Duncan's multiple range test.
